# Impact of Nano-Bromocriptine on Egg Production Performance and Prolactin Expression in Layers

**DOI:** 10.3390/ani11102842

**Published:** 2021-09-29

**Authors:** Ahmed Dawod, Noha Osman, Hanim S. Heikal, Korany A. Ali, Omaima M. Kandil, Awad A. Shehata, Hafez M. Hafez, Hamada Mahboub

**Affiliations:** 1 Department of Husbandry and Animal Wealth Development, Faculty of Veterinary Medicine, University of Sadat City, Menofia 32897, Egypt; adawod@vet.usc.edu.eg (A.D.); noha.osman@vet.usc.edu.eg (N.O.); hanem.hekal@vet.usc.edu.eg (H.S.H.); hamada.mahboub@vet.usc.edu.eg (H.M.); 2 Center of Excellence for Advanced Science, Advanced Materials and Nanotechnology Group, Applied Organic Chemistry Department, National Research Centre, Dokki, Giza 12622, Egypt; kornykhlil@gmail.com; 3 Center of Excellence for Embryo and Genetic Resources Conservation Bank, Department of Animal Reproduction and Artificial Insemination, Veterinary Research Division, National Research Center, Dokki, Giza 12622, Egypt; omaima_mk@yahoo.com; 4 Avian and Rabbit Diseases Department, Faculty of Veterinary Medicine, University of Sadat City, Menofia 32897, Egypt; Awad.Shehata@pernaturam.de; 5 Research and Development Section, PerNaturam GmbH, 56290 Gödenroth, Germany; 6 Institute of Poultry Diseases, Free University Berlin, 14195 Berlin, Germany

**Keywords:** prolactin, sodium alginate, nanotechnology, egg production, qPCR, chickens

## Abstract

**Simple Summary:**

Egg production is one of the most vital axes in the poultry industry. During the late laying period, the egg production continuously decreases, and pauses among the sequence of egg laying increases; however, the feed costs remain constant. Several attempts were carried out to improve the reproductive performance of laying hens by decreasing the prolactin level in the blood; an increase in this hormone initiates the onset of incubation behavior in chickens. In this study, we investigated the potential use of nano-bromocriptine to the improve egg production performance in laying hens. The use of alginate-bromocriptine leads to a significant reduction in the prolactin expression in the pituitary gland, which in turn allows the elongation in sequences and reduction in pauses, as well as the feed per dozen egg in laying hens. Further studies are needed to assess the impacts of nano-bromocriptine on other performance parameters. Thus, the improvement of egg production persistency must also go hand in hand with sustainable egg quality and the maintenance of the birds’ health.

**Abstract:**

The current study aimed to investigate the potential use of nano-bromocriptine in improving the laying performance of late laying hens by modulating the prolactin gene expression. A total of 150 NOVOgen brown laying hens aged 70 weeks were randomly allocated into three groups of 50 birds each. The first group was kept as a control, while the second and the third groups were treated with bromocriptine and nano-bromocriptine, respectively, at a dose of 100 µg/kg body weight per week. The pause days, egg production, feed per dozen egg, and Haugh unit were determined on a monthly basis. Also, the relative prolactin gene expression in the pituitary gland was quantified using qPCR and the number of the ovarian follicles was determined after slaughtering at the 84th week of age. It was found that nano-bromocriptine and bromocriptine improved egg laying performance with minimal pause days, reduced feed per dozen egg, and depressed the relative prolactin gene expression; however, nano-bromocriptine treatment was significantly effective compared to bromocriptine. In conclusion, nano-bromocriptine might be beneficial for elongating sequences and reducing pauses.

## 1. Introduction

Although the global table eggs production has increased over the past decade to 76.7 million tonnes in 2018 [1], further improvement of egg production performance is urgently needed to fulfill the high demand for animal proteins. This highlights the urgent need to keep the laying persistency with sustainable egg production and quality that goes hand in hand with maintaining health and animal welfare, by considering the bird’s physiology, nutritional requirements, management system, reproductive status, and breed selection. Although the extreme efforts which have been done to keep the persistency of egg production, a reduction in egg production accompanied by a deterioration of egg quality are usually common at or around 72 weeks of age [2]. During this period, the egg production continuously decreased and pauses among the sequence of egg laying increased with constant feed costs, causing huge economic losses.

Prolactin belongs to adenohypophysis hormones, is one of the most blamed factors accompanied by a progressive reduction of egg laying performance during the late laying period. This hormone is progressively increased by the time in plasma of late laying hens. It prevents gonadotrophin, which stimulates ovulation as well as estrogen production at the ovarian level [3]. The ovarian tissues in laying hens targeted the prolactin as it expressed the prolactin receptor mRNA [4]. So far, prolactin inhibits estradiol secretion in white ovarian follicles. However, it may improve or depress steroidogenesis in yellow ovarian follicles depended upon the level of prolactin dose, the type of the follicular layer secreting steroids, the order of the ovarian follicle in the ovulation hierarchy, and the phase of the ovulation cycle [5]. Increasing prolactin level during the active stage of laying is accompanied by the appearance of broodiness and cessation of egg production [6]. Several attempts were carried out to improve the reproductive performance of laying hens via decreasing prolactin using recombinant-derived chicken prolactin fusion protein or anti-vasoactive intestinal polypeptide serum to prevent broodiness in laying hens [7,8]. Others tried to depress prolactin chemically using a dopamine agonist, bromocriptine, an ergot derivative [9,10,11]. The release of prolactin from the adenohypophysis is governed by dopamine as it prevents the stimulatory effect of the vasoactive intestinal peptide on prolactin secretion. Therefore, bromocriptine as a dopamine agonist could be used to overcome the broodiness behaviors in laying hens [8]. Indeed, subcutaneous injection of bromocriptine in laying hens during the 17th to 36th week of life increases the egg production and depresses the prolactin production during the laying cycle up to the 72nd week of age [3]. 

Nevertheless, due to the low bioavailability of bromocriptine [12], there is a great demand for more convenient, effective, and safe ways for drug delivery. Nano-drug delivery systems could be a promising alternative to extend the half-life time of active principles and to sustain its delivery to the target sites [12,13,14,15,16]. It was found that nanocomposites reduced the dose of the drug and the desired biological activity could be obtained with minimal side effects. Therefore, the present study aimed to synthesize, and characterize, alginate-bromocriptine nanocomposite as well as to assess its efficacy on egg laying performance during the late laying stage. Moreover, its modulatory effect on the prolactin hormone gene expression in the pituitary gland was studied.

## 2. Materials and Methods

### 2.1. Preparation of Alginate-Bromocriptine Nano-Composite

The alginate-bromocriptine nano-composite was prepared according to Siddique et al. [12]. Briefly, 100 mg of 2-Bromo-α-ergocryptine (Sigma-Aldrich, St. Louis, MO, USA) was dissolved in 5 mL ethanol and added portion wise to sodium alginate solution, previously prepared by dissolving 0.1 g of sodium alginate (Fisher Scientific, Waltham, MA, USA) in 100 mL distilled water with stirring. The addition process was performed drop by drop over 30 min with stirring and worming at 40 °C. The mixture was then heated at 50 °C under ultra-sonication at 100 W with 35 kHz for 45 min. The obtained nano-composite solution was air-dried then stored at 4 °C, dry, and dark conditions. 

The formation and the morphology of alginate-bromocriptine nano-composite were characterized by transmutation electron microscopy (TEM, JEM-2100, JEOL- Tokyo Japan) and the average size of the prepared nano-composite was estimated. The Fourier Transform Infrared Spectrophotometer (FTIR) spectra were analyzed using a spectrometer (JASCO, Tokyo, Japan) in the range of 400–4000 cm^−1^. Potassium bromide discs (KBr) (5 mg of particles, 100 mg KBr pellets) were used as reference material. The optical properties of the bromocriptine and alginate-bromocriptine nanocomposite were investigated by measuring the UV-visible spectrophotometer (JASCO spectrophotometer, Tokyo, Japan) in the range of 200–800 nm. 

### 2.2. Experimental Assessment of the Efficacy of Bromocriptine and Alginate-Nano-Bromocriptine

#### 2.2.1. Ethical Approval

All procedures of the experiment were conducted under the ethical guidelines of the Institutional Animal Care and Use Committee (IACUC), Faculty of Veterinary Medicine, University of Sadat City, Egypt (Ethical approval number: VUSC-017-1-19).

#### 2.2.2. Experimental Design

One hundred fifty laying hens of NOVOgen brown strain of 70 weeks age were selected from a local commercial layer farm in Egypt based on good feathers, body weight (mean = 1800 ± 150 g), and free from any abnormalities. Birds were randomly allocated into 3 groups of 50 birds per each. Hens were allowed to have 2 weeks acclimatization period. Chickens in the first group were treated with saline and kept as control, while chickens kept in the second and third groups were treated with bromocriptine (2-bromo-alpha-ergocriptine, Sigma-Aldrich, St. Louis, MO, USA) and the prepared nano-bromocriptine, respectively, at a dose of 100 µg/kg BW/week according to Reddy et al. [17] for 12 weeks (from 72nd to 84th week of age). Each group was subdivided into two subgroups; the first subgroup was treated orally, whereas the second subgroup was treated subcutaneously underneath the wing. Hens were kept under the standard laying conditions on the litter floor system of sawdust equipped with automatic drinker and feeding systems. The feed containing 16% crude protein (corn-soybean meal of 16% CP, 2900 kcal ME/kg diet) and 120 g/hen/day was used, fresh-water give at ad libitum. The photoperiod was kept at 16 h of light/day, achieved via an automatic lighting system. The temperature of the house was kept at 24 ± 3 °C.

#### 2.2.3. Egg Production Performance 

Eggs were collected and recorded daily from each group at 12 AM. Pause days, egg production%, feed intake, and feed per dozen eggs were daily evaluated for each subgroup. Pause days were daily estimated for each laying hen as the hens were separately reared in deep litter system. Also, twelve eggs were randomly selected from each group/subgroup at 76th, 80th, 84th / week of age to determine the Haugh unit according to Haugh [18] using the formula: Haugh unit $\left(HU\right)=100\times log\;\left(H-1.7W^{0.37}+7.6\right)$, where “*H*” is the albumen height and “*W*” is the egg weight. 

At the 84th week of age, all hens were slaughtered by manual cervical dislocation then dressed. The ovary was investigated to estimate the number of normal large yellow follicles (LYF) (>10 mm diameter), small yellow follicles (SYF) (5–10 mm diameter), and large white follicles (LWF) (3–5 mm diameter) according to Renema et al. [19]. 

#### 2.2.4. Prolactin Gene Expression in Pituitary Gland Using qRT-PCR

Just after slaughtering, the pituitary gland was collected randomly from 5 hens/subgroup. The RNA extraction was carried out using a TRIzol reagent (Invitrogen, Carlsbad, CA, USA) according to the manufacturer and the cDNA was formed from total RNA using Maxima First Strand cDNA Synthesis Kit (Life Technologies, Carlsbad, CA, USA). The PRLE2F: 5′-GTTTGTTTCTGGCGGTTC-3′, and PRLE2R: 5′-AAATTCATTGAATATTTCTGAAG-3′ primers were used to amplify 181 bp fragment of prolactin gene [20], while the QGAPDHF: 5′-CTGCCGTCCTCTCTGGC-3′ and QGAPDHR: 5′-GACAGTGCCCTTGAAGT GT-3′ primers were used as an internal control to amplify 119 bp fragment of glyceraldehyde 3-phosphate dehydrogenase gene [21]. The qPCR was performed in duplicate in a final volume of 25 µL nuclease-free water containing 12.5 µL of Platinum SYBR Green qPCR supermix, 0.05 µL of ROX reference dye (Invitrogen, Carlsbad, CA, USA), 0.2 µM of each primer, 2 µL of cDNA using a 7500 Fast Real-Time PCR System (Applied Biosystems, Foster City, CA, USA). The cycler program was 1× 95 °C/10 min, and 40× (95 °C/30 s, 57 °C/1 min and 72 °C for 30 s). The melting curve of a single peak was determined and used for the gene expression analysis. The normalization was done using the GAPDH as an endogenous control. The relative quantification was carried out using the $2^{-\Delta \Delta \mathrm{CT}}$ method [22].

### 2.3. Statistical Analysis

The data were collected and enrolled into statistical analysis using SAS software (SAS User’s Guide: Statistics, Version 8.1 Edition, 2000, SAS Inst. Inc., Cary, NC, USA). Percentage data were subjected to arcsine transformation. Then the data were analyzed using two ways ANOVA of the General Linear Models (GLM) procedures of SAS software according to the following statistical model:

$$Y_{\mathrm{ijk}}=\;\mu +\alpha_{i}+\beta_{j}+{\left(\alpha \beta \right)}_{\mathrm{ij}}+\epsilon_{\mathrm{ijk}}$$

where, $Y_{\mathrm{ijk}}$ = overall observation, μ = overall mean, $\alpha_{i}$ was the treatment effect i = 1, 2, 3 for control, bromocriptine, and nanobromocriptine treatments, $\beta_{j}$ was the route of administration j = 1, 2 for oral, and injection rout of administrations, ${\left(\alpha \beta \right)}_{\mathrm{ij}}$ was the interaction between treatments and route of administration and $\epsilon_{\mathrm{ijk}}$ was the random error. Tukey test was used as mean separation test and results were expressed as least square means ± SE. The level of significance was seated at (*p* < 0.05).

## 3. Results

### 3.1. Analysis of Alginate-Bromocriptine

**Figure 1:**
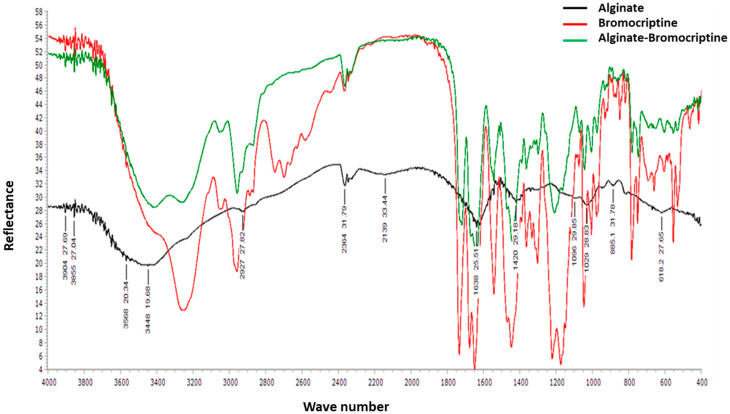
Fourier transform infrared (FTIR) spectra of alginate bromocriptine.

The FTIR spectroscopy of bromocriptine and alginate-bromocriptine composite gave information about chemical bonding to confirm the purity and the formation of the alginate-bromocriptine nanocomposite. The FTIR spectrum displayed bands at 3500 cm^−1,^ corresponding to the OH bending of the hydroxyl group. The stretching vibration appeared as bands at 2300 cm^−1^, 1500 cm^−1^, and 1200 cm^−1^, and are attributed to -CO, C=O, and C-O-C bonds, respectively. The FTIR spectroscopy pattern of the alginate-bromocriptine nanocomposite revealed a band at 700 cm^−1^, corresponding to -CH bending; this confirms a bond formation between alginate and bromocriptine (Figure 1).

**Figure 2:**
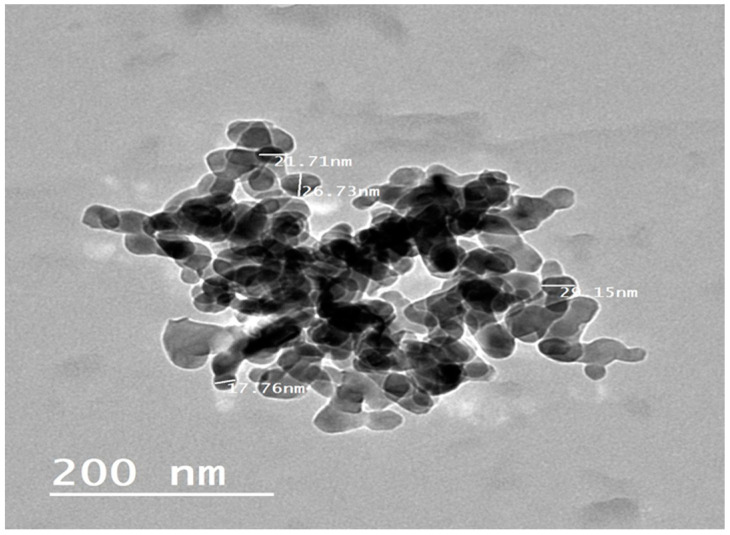
Transmission electron microscope image of alginate-bromocriptine nanocomposite with scale 200 nm at 25 °C.

The TEM analysis of the prepared alginate-bromocriptine nanocomposite is shown in Figure 2. The alginate-bromocriptine nanocomposite particles are semi-spherical in shape with a size of 20 to 36 nm, and uniformly distributed. The average particle size is up to 33 nm.

**Figure 3:**
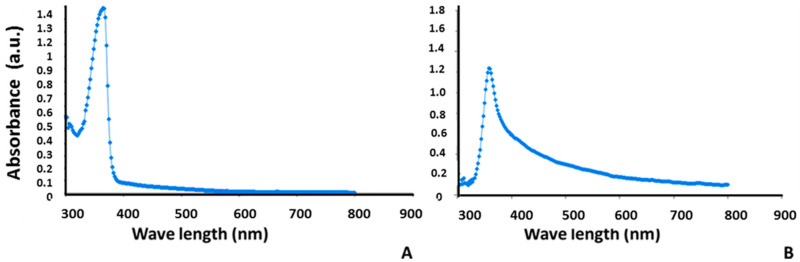
UV-visible absorption spectroscopy for bromocriptine (**A**) and alginate-bromocriptine nanocomposite (**B**).

The optical properties of the bromocriptine and alginate-bromocriptine nanocomposite measured in the wavelength range of 300–900 nm showed a strong absorption band at 366 nm, corresponding to the pure bromocriptine, in addition to a low absorption band at the visible region (Figure 3A,B). Alginate-bromocriptine nanocomposite did not show a significant shift where the strong absorption band appeared at 362 nm. These results revealed a decrease in the bandgap due to the crystallite size of the prepared nanoparticles.

### 3.2. Assessment of the Efficacy of Bromocriptine and Nano-Bromocriptine on Egg Production Performance

#### 3.2.1. Pause Days, Egg Production Percentage, and Feed per Dozen Egg

The total number of pause days routinely decreased in nano-bromocriptine treated chickens compared to those treated with bromocriptine and control. This trend appeared clearly over the entire experimental period. The lowest numbers of pause days were 5.38 ± 0.33, 5.93 ± 0.26, and 7.27 ± 0.30 days in chickens treated with alginate-bromocriptine nanocomposite at the 76th, 80th, and 84th week of age, respectively. Furthermore, the oral administration showed the best pause days compared to the injection route. Thus, the lowest number of pause days in the hens treated orally with alginate-bromocriptine nanocomposite were 3.93 ± 0.35, 5.29 ± 0.35, and 6.46 ± 0.44 days at the 76th, 80th, and 84th week of age, respectively (Table 1).

The nano-bromocriptine treated hens recorded the highest egg production percentage (78.33 ± 1.26 and 74.54 ± 1.16) at the 76th and 80th week of life, respectively, compared to the bromocriptine treated (71.55 ± 1.25 and 71.11 ± 1.11%) or control (61.42 ± 0.95% and 61.9 ± 0.78) hens (Table 1). However, at the 84th week of age, both nano-bromocriptine (68.13 ± 1.45) and bromocriptine (65.54 ± 1.71%) treated hens sustained a higher egg production percentage compared to the non-treated hens (51.16 ± 1.42%). On the other hand, the oral administration of nano-bromocriptine sustained the highest egg production percentage (84.26 ± 1.28, 78.80 ± 1.34, and 72.52 ± 1.92, at 76th, 80th, and 84th week-old, respectively).

#### 3.2.2. Feed Consumption per Dozen Egg, and Haugh Unit

The feed per dozen egg was significantly varied among different bromocriptine forms and control groups (Table 2). Both the bromocriptine and nano-bromocriptine treated hens exhibited a significant reduction in feed per dozen egg compared to the control group over the entire experimental period (*p* < 0.0001). Alginate-bromocriptine nanocomposite treatment significantly reduced the feed per dozen egg (1.87 ± 0.03 and 1.96 ± 0.03 kg, respectively) at the 76th and 80th week of age, followed by bromocriptine treatment (2.06 ± 0.05 and 2.05 ± 0.03 kg, respectively) and control (2.38 ± 0.04 and 2.35 ± 0.03 kg, respectively). The birds that received oral nano-bromocriptine sustained the lowest feed per dozen egg values (1.72 ± 0.03; 1.84 ± 0.03; 2.02 ± 0.06 kg at the 76th, 80th, and 84th week of age, respectively) compared to the other administration methods.

The Haugh unit possessed no significant differences among different treatments at the 76th and 80th week of age (Table 2). In contrast, the Haugh unit at the 84th week of age was significantly (*p* < 0.0007) increased in the non-treated birds (83.06 ± 1.16) compared to both the bromocriptine (78.99 ± 1.16) and nano-bromocriptine (76.82 ± 1.08) treated groups.

#### 3.2.3. Ovarian Follicles, and Prolactin Gene Expression in the Pituitary Gland Tissue

The number of different types of ovarian follicles varied according to the use of alginate-bromocriptine nanocomposite or bromocriptine during the late laying phase (Table 3). The groups treated with bromocriptine and nano-bromocriptine showed significantly higher numbers of LYF (5.9 ± 0.19, and 6.3 ± 0.15, respectively) compared to the control group (5.33 ± 0.2). However, a non-significant increase in SYF and decrease in LWF were found in chickens treated with nano-bromocriptine.

**Figure 4:**
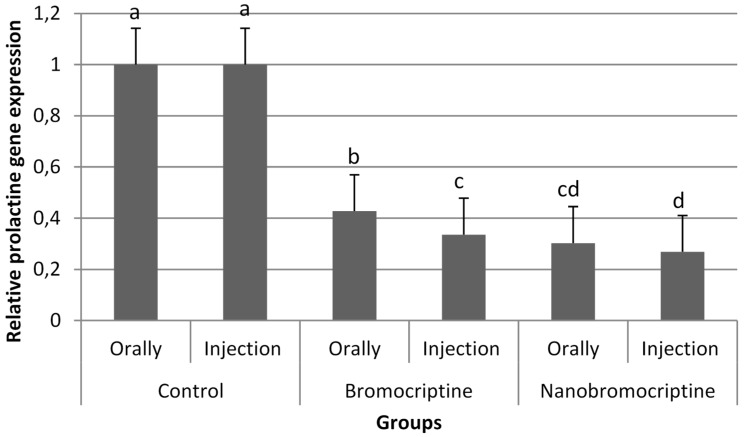
Quantitative gene expression of prolactin gene in pituitary after either bromocriptine or nano-bromocriptine administration. The qRT-PCR data were normalized relative to the expression of glyceraldehyde-3-phosphate dehydrogenase (GAPDH) as endogenous control and calculated using the $2^{-\Delta \Delta \mathrm{CT}\;}$ method (*n* = 5). Different superscripts indicated significant differences at *p* < 0.05. Error bars indicate the standard error of the mean.

Additionally, the expression of the prolactin gene was determined in the pituitary gland tissues of late laying hens (Figure 4). The findings of the prolactin gene expression showed obvious depression in response to the alginate-bromocriptine nanocomposite treated group. Both bromocriptine and nano-bromocriptine exhibited a significant depression of prolactin gene expression in treated birds. Furthermore, the findings revealed that the depression of prolactin gene expression was enhanced via the injection route of administration in bromocriptine-treated birds (*p* < 0.05). However, there were no significant differences between the two routes in the groups treated with nano-bromocriptine.

## 4. Discussion

Eggs are important food for the human population because of their inexpensive price and high nutrient value. There is a huge demand for animal protein supply that could be covered with eggs [23]. In the present investigation, we tried to modulate the level of the prolactin gene expression in hens during their late laying phase using a minimal dose of bromocriptine and nano-bromocriptine. 

The alginate nanocomposites have recently received much attention as drug delivery nanoparticles [14]. It has efficient biodegradability, biocompatibility, and mucoadhesiveness nature making them superior natural polymers that are ready to use as a drug delivery nanocomposite for target tissue [24]. The formulation used is highly advantageous for maximizing the effectiveness of the drug on target tissues. In our study, the alginate-bromocriptine nano-composite was synthesized and characterized. The prepared particle size ranged from 20 to 36 nm. The size of nanoparticles is a critical criterion for the crossing of the mucosal tissue barriers and the enhancement of the cellular uptake [25]. The size of most nanoparticles ranged between 50–250 nm [26,27]. However, the average size of alginate-bromocriptine nanocomposite could reach 20 nm [12]. The biological activity of bromocriptine and the prepared nano-bromocriptine was carried out in the 70 week old NOVOgen brown hens. Our findings revealed that alginate-bromocriptine nano-composite improved the egg laying performance in late laying hens, thus treated hens sustained the highest egg production, the lowest pause days, and the lowest feed per dozen egg compared to the negative control and bromocriptine treated birds. This could be due to rapid gut absorption and bioavailability of the alginate-bromocriptine nano-composite compared to bromocriptine. It was reported that nano-drugs could be accumulated in the target tissue with longer blood circulation time and binding properties [28]. Moreover, the drug nano-particles crossed efficiently from blood vessels and lymphatics. Furthermore, nano-drugs had positive charges that could be attracted to the negative charges of mucin, this property may improve the transportation and absorption of the drug through the epithelial membranes [29]. 

Furthermore, bromocriptine increased significantly egg laying performance compared with control, in terms of egg production percentage, reduction of pause days, and feed per dozen egg. The same findings were proved in earlier studies [3,10,11,17,30,31]. This could be due to the lower circulating prolactin in the bromocriptine and alginate-bromocriptine nanocomposite treated hens, thus the increase of prolactin above its physiological range resulting in a subsequent decrease in circulating gonadotropins, regression of the ovarian functions, ending of the reproductive phase of the laying hens, and altered it to the brooding phase [32]. However, there is one significant limitation in this study: it was not possible to determine both plasma level of luteinizing hormone (LH) and sex steroids.

Interestingly, significant increases in LYF numbers were reported in bromocriptine and alginate-bromocriptine nanocomposite treated birds. This may explain the higher egg production percentages that were observed in these groups. These findings could be attributed to the low level of the circulating prolactin hormone in treated birds. Prolactin depressed the development of ovarian follicles to reach the final stage through interference with the follicular steroidogenesis in avian species [33]. However, the outcomes suggested that the Haugh unit was significantly decreased in response to bromocriptine and alginate-bromocriptine nanocomposite treatment. In contrast, Banu et al. reported that bromocriptine treatment did not affect the Haugh unit of the eggs of laying hens [31]. This outcome may belong to high egg production sustained in such groups compared with control.

Oral administration may enhance the alginate-bromocriptine nanocomposite effects. This could be due to the high absorption of alginate-bromocriptine nanocomposite particles in the chicken gut compared with bromocriptine. Caster et al. found that oral and intravenous routes were the most common routes of administration of the nano-drugs rather than the transdermal route [34]. Furthermore, the oral administration of bromocriptine is rapidly and incompletely absorbed in animals. However, this rapidly absorbed portion is highly metabolized in the liver, so its bioavailability is rapidly declined [35]. 

Nano-drug had many advantages properties, such as improved solubility, efficacy, tissue selectivity, and reduced toxicity compared to the conventional drugs [34,36,37,38]. This could explain why the alginate-bromocriptine nanocomposite depressed the relative prolactin gene expression in the pituitary gland compared with bromocriptine. Moreover, the injectable route of administration of bromocriptine sustained lower prolactin gene expression compared with the oral route. Linearly, it was reported that injection of bromocriptine frequently induced a prolonged normoprolactinemia compared to oral administration [39]. In contrast, there were no significant differences between the two different routes of alginate-bromocriptine nanocomposite administration, highlighting the high absorption and bioavailability of nanoform. 

## 5. Conclusions

Nano-bromocriptine could be used to improve the egg production performance in late laying hens. The same effects were obtained with bromocriptine but with lower efficacy than its nanoform. Further studies about its impact on other performance and health parameters such as carcass quality, blood parameters, LH plasma concentration and sex steroids as well as liver functions are in progress.

## Figures and Tables

**Table 1:** Effects of bromocriptine and nano-bromocriptine on pause days and egg production percent of late laying hens.

Parameter		Pause Days (Day)			Egg/Hen/D (%)		
		76th Week	80th Week	84th Week	76th Week	80th Week	84th Week
Treatment	Control (*n =* 50)	11.07 ± 0.26 ^a^	10.00 ± 0.25 ^a^	11.56 ± 0.34 ^a^	61.42 ± 0.95 ^c^	61.90 ± 0.78 ^c^	51.16 ± 1.42 ^b^
	Bromocriptine (*n =* 50)	7.05 ± 0.32 ^b^	6.89 ± 0.28 ^b^	8.20 ± 0.35 ^b^	71.55 ± 1.25 ^b^	71.11 ± 1.01 ^b^	65.54 ± 1.71 ^a^
	Nano-bromocriptine (*n =* 50)	5.38 ± 0.33 ^c^	5.93 ± 0.26 ^c^	7.27 ± 0.30 ^c^	78.33 ± 1.26 ^a^	74.54 ± 1.16 ^a^	68.13 ± 1.45 ^a^
Administration	Orally (*n =* 75)	7.14 ± 0.39 ^b^	7.15 ± 0.32 ^b^	8.94 ± 0.36	73.37 ± 1.26 ^a^	71.04 ± 1.12 ^a^	62.41 ± 1.62 ^a^
	Injection (*n =* 75)	8.52 ± 0.32 ^a^	8.06 ± 0.24 ^a^	9.08 ± 0.31	67.49 ± 1.04 ^b^	67.33 ± 0.78 ^b^	60.82 ± 1.44 ^a^
Treatment χ administration	Control (oral, *n =* 25)	11.11 ± 0.37 ^a^	10.07 ± 0.37 ^a^	11.50 ± 0.48 ^a^	61.15 ± 1.34 ^d^	61.56 ± 1.12 ^c^	51.65 ± 1.99 ^c^
	Control (injection, *n =* 25)	11.04 ± 0.38 ^a^	9.93 ± 0.35 ^a^	11.63 ± 0.48 ^a^	61.69 ± 1.37 ^d^	62.25 ± 1.09 ^c^	50.67 ± 2.05 ^c^
	Bromocriptine (oral, *n =* 25)	6.39 ± 0.37 ^c^	6.11 ± 0.44 ^c^	8.86 ± 0.53 ^b^	74.71 ± 1.50 ^b^	72.75 ± 1.81 ^b^	63.04 ± 2.62 ^b^
	Bromocriptine (injection, *n =* 25)	7.71 ± 0.49 ^b^	7.68 ± 0.28 ^b^	7.54 ± 0.42 ^c,d^	68.39 ± 1.87 ^c^	69.46 ± 0.84 ^b^	68.05 ± 2.12 ^a,b^
	Nano-bromocriptine (oral, *n =* 25)	3.93 ± 0.35 ^d^	5.29 ± 0.35 ^c^	6.46 ± 0.44 ^d^	84.26 ± 1.28 ^a^	78.80 ± 1.34 ^a^	72.52 ± 1.92 ^a^
	Nano-bromocriptine (injection, *n =* 25)	6.82 ± 0.41 ^c^	6.57 ± 0.36 ^c^	8.07 ± 0.34 ^b,c^	72.40 ± 1.58 ^b,c^	70.29 ± 1.55 ^b^	63.75 ± 1.78 ^b^
*p*-value	Treatment	<0.0001	<0.0001	<0.0001	<0.0001	<0.0001	<0.0001
	Administration	<0.0001	<0.0026	<0.7091	<0.0001	<0.0007	<0.3572
	Interaction	<0.0014	<0.0425	<0.0062	<0.0003	<0.0032	<0.0052

Values are presented as least square mean ± SE. ^a–d^ Means within the same column for each parameter with different superscripts are statistically different at *p* < 0.05 (Two-way ANOVA, Tukey post-hoc test).

**Table 2:** Effects of bromocriptine and nano-bromocriptine on feed consumption per dozen egg and Haugh unit of late laying hens.

Parameter		Feed/Dozen Egg (kg)			Haugh Unit		
		76th Week	80th Week	84th Week	76th Week	80th Week	84th Week
Treatment	Control (*n =* 50)	2.38 ± 0.04 ^a^	2.35 ± 0.03 ^a^	2.94 ± 0.11 ^a^	76.70 ± 0.53	77.01 ± 1.19	83.06 ± 1.16 ^a^
	Bromocriptine (*n =* 50)	2.06 ± 0.05 ^b^	2.05 ± 0.03 ^b^	2.29 ± 0.08 ^b^	75.63 ± 1.74	77.29 ± 1.39	78.99 ± 1.16 ^b^
	Nano-bromocriptine (*n =* 50)	1.87 ± 0.03 ^c^	1.96 ± 0.03 ^c^	2.16 ± 0.05 ^b^	76.24 ± 1.60	75.20 ± 0.84	76.82 ± 1.08 ^b^
Administration	Orally (*n =* 75)	2.02 ± 0.04 ^b^	2.07 ± 0.03 ^b^	2.44 ± 0.08 ^a^	75.12 ± 0.58	76.70 ± 0.90	79.21 ± 0.92
	Injection (*n =* 75)	2.18 ± 0.04 ^a^	2.16 ± 0.03 ^a^	2.48 ± 0.07 ^a^	77.26 ± 1.52	76.30 ± 1.03	80.04 ± 0.93
Treatment χ administration	Control (oral, *n =* 25)	2.39 ± 0.05 ^a^	2.36 ± 0.04 ^a^	2.91 ± 0.15 ^a^	76.83 ± 0.72	77.99 ± 2.07	83.29 ± 1.58 ^a^
	Control (injection, *n =* 25)	2.37 ± 0.05 ^a^	2.33 ± 0.04 ^a^	2.96 ± 0.16 ^a^	76.58 ± 0.88	76.02 ± 0.80	82.83 ± 1.70 ^a,b^
	Bromocriptine (oral, *n =* 25)	1.95 ± 0.04 ^b,c^	2.02 ± 0.05 ^b^	2.41 ± 0.14 ^b^	74.50 ± 0.85	75.75 ± 1.63	75.28 ± 1.77 ^b,c^
	Bromocriptine (injection, *n =* 25)	2.17 ± 0.08 ^b^	2.08 ± 0.03 ^b^	2.17 ± 0.08 ^b,c^	76.76 ± 3.49	78.83 ± 2.19	82.71 ± 1.49 ^a^
	Nano-bromocriptine (oral, *n =* 25)	1.72 ± 0.03 ^c^	1.84 ± 0.03 ^c^	2.02 ± 0.06 ^c^	74.04 ± 1.08	76.35 ± 0.92	79.06 ± 1.41 ^b,c^
	Nano-bromocriptine (injection, *n =* 25)	2.02 ± 0.05 ^b^	2.08 ± 0.05 ^b^	2.30 ± 0.07 ^b,c^	78.44 ± 2.81	74.05 ± 1.28	74.58 ± 1.64 ^c^
*p*-value	Treatment	<0.0001	<0.0001	<0.0001	<0.8726	<0.3834	<0.0007
	Administration	<0.0002	<0.0079	<0.7266	<0.2117	<0.7665	<0.5290
	Interaction	<0.0083	<0.0066	<0.0473	<0.5373	<0.1923	<0.0013

Values are presented as least square mean ± SE. ^a–c^ Means within the same column for each parameter with different superscripts are statistically different at *p* < 0.05 (Two-way ANOVA, Tukey post-hoc test).

**Table 3:** Effects of bromocriptine and nano-bromocriptine on ovarian follicles of late laying hens.

Parameter		Ovarian Follicles		
		LYF	SYF	LWF
Treatment	Control (*n =* 50)	5.33 ± 0.20 ^b^	5.85 ± 0.63	8.93 ± 1.20
	Bromocriptine (*n =* 50)	5.90 ± 0.19 ^a^	6.40 ± 1.37	6.80 ± 0.81
	Nano-bromocriptine (*n =* 50)	6.30 ± 0.15 ^a^	8.10 ± 0.95	6.80 ± 0.88
Administration	Orally (*n =* 75)	5.87 ± 0.13	6.73 ± 1.02	7.33 ± 0.87
	Injection (*n =* 75)	5.82 ± 0.19	6.83 ± 0.68	7.68 ± 0.75
Treatment χ administration	Control (oral, *n =* 25)	5.40 ± 0.27	6.20 ± 0.97	8.60 ± 1.66
	Control (injection, *n =* 25)	5.25 ± 0.31	5.50 ± 0.87	9.25 ± 1.97
	Bromocriptine (oral, *n =* 25)	6.00 ± 0.21	6.60 ± 2.64	6.60 ± 1.60
	Bromocriptine (injection, *n =* 25)	5.80 ± 0.33	6.20 ± 1.20	7.00 ± 0.63
	Nano-bromocriptine (oral, *n =* 25)	6.20 ± 0.13	7.40 ± 1.69	6.80 ± 1.39
	Nano-bromocriptine (injection, *n =* 25)	6.40 ± 0.27	8.80 ± 0.97	6.80 ± 1.24
*p*-value	Treatment	<0.0021	<0.3429	<0.2705
	Administration	<0.8141	<0.9385	<0.7695
	Interaction	<0.6965	<0.7694	<0.9749

LYF: number of normal large yellow follicles (>10 mm diameter); SYF: number of small yellow follicles (5–10 mm diameter); LWF: number of large white follicles (3–5 mm diameter). Values are presented as least square mean ± SE. ^a–b^ means within the same column for each parameter with different superscripts are statistically different at *p* < 0.05 (Two-way ANOVA, Tukey post-hoc test).

## Data Availability

The data sets used during the current study are available from the corresponding author on reasonable request.
